# The gender-specific impact of starvation on mitotypes diversity in adults of *Drosophila melanogaster*

**DOI:** 10.1098/rsob.220108

**Published:** 2022-09-28

**Authors:** Tao Wang, Tian-Chu Li, Yun-Heng Miao, Luo-Nan Wu, Yu-Qiao Chen, Da-Wei Huang, Jin-Hua Xiao

**Affiliations:** College of Life Sciences, Nankai University, Tianjin 300071, People's Republic of China

**Keywords:** starved, mitochondria, genetic diversity, fruit fly

## Abstract

In animals, starvation can increase the level of reactive oxygen species (ROS) in some tissues. Mitochondrial DNA (mtDNA) is more vulnerable to being attacked by ROS due to the lack of histone protection, leading to oxidative damage. However, whether starvation is associated with the genetic diversity of mtDNA remains unclear. Here, by using adult individuals of *Drosophila melanogaster* under three different feeding treatments (starvation, with the provision of only water, and normal feeding), based on the high-throughput sequencing results of the PCR amplicons of the partial sequences of the mitochondrial gene cytochrome c oxidase subunit I (*mt-cox1*), no significant difference in the mean number of mitochondrial haplotypes and the mean genetic distance of haplotypes within individuals were identified between the three treatment groups. Coupled with the low proportion of heterogeneous *mt-cox1* sequences within each individual, it suggested that starvation had a limited impact on mitotype genetic diversity and mitochondrial function. Nevertheless, starvation could significantly increase the sequence number of haplotypes containing specific mutations, and for males with higher levels of mitochondrial heteroplasmy than females in the normal feeding group, starvation could further increase their mitochondrial heteroplasmy.

## Graphical abstract

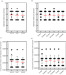

## 1. Introduction

Under certain circumstances, the living environment of animals can be characterized by periods of food shortage or even foodlessness, when the metabolism of the organisms will undergo great changes, such as upregulation of catabolism and downregulation of anabolism [1–3]. In *Drosophila melanogaster*, starvation causes hyperactivity and sleep suppression physiologically [4–7]. Moreover, sleep loss can increase the content of mitochondrial reactive oxygen species (mtROS) in the dorsal fan-shaped body neurons of the *Drosophila* brain, consequently altering the redox state of neurons [8]. Meanwhile, the gut also experiences a large accumulation of reactive oxygen species (ROS), which will further trigger oxidative stress to hasten the death of fruit flies [9]. Mitochondria are the primary source of intracellular ROS [10–12], and mitochondrial DNA (mtDNA) is a circular double-stranded molecule that forms nucleoids (nucleoprotein complexes/structures) in mammalian mitochondria rather than being ‘naked’ [13,14]. Although mitochondria have antioxidant systems to reduce the damage from mtROS [15] and mtDNA can form nucleoids, mtDNA is still highly sensitive to mtROS, making it more vulnerable to being attacked by mtROS, resulting in oxidative damage [16].

There is not only one type of mtDNA in an organism, but actually, the same individual may contain two or more types of mtDNA, which is known as mitochondrial heteroplasmy [17,18]. The types of mitochondrial heteroplasmy can be classified as site heteroplasmy and length heteroplasmy, which are usually derived from somatic mutations that occur mostly in embryonic stages [19], paternal leakage, maternally transmitted heteroplasmy [20], doubly uniparental inheritance in the bivalve [21,22], etc. In *Drosophila*, some species (*D*. *melanogaster*, *D*. *simulans*, *D*. *mauritian* and *D*. *subobscura*) were detected to possess mitochondrial length heteroplasmy through the method of restriction digestion [23,24]. By cytoplasmic microinjection of different mitotypes to construct mitochondrial heteroplasmic flies, it was discovered that the selection of mitotypes within individuals was non-random [25]. In addition, ageing and mating can also affect mitochondrial heteroplasmy [26]. In the past decade, the research on mitochondrial heteroplasmy in fruit flies has focused more on the mechanisms of paternal leakage origin [27–30], evolution [31,32] and elimination [33,34].

DNA barcoding has been widely used since it was proposed in 2003 [35]. With the accumulation of barcode sequences, some databases such as NCBI and BOLD system [36] have recorded a large number of species barcodes, among which the commonly used DNA barcode in animals is the 5′ end region of the mitochondrial *cox1* gene [37–39], particularly in insects [40]. Currently, the high-throughput *cox1* amplicon sequencing can be used for rapid identification of members in ecological communities [41], determination of animal diets [42,43], biodiversity surveys [40,44], discovery of cryptic species [45] and conservation biology [46], along with the estimation of intraspecific genetic diversity [47]. According to the sequencing principle, *cox1* amplicon sequencing can also be used to study intra-individual genetic diversity, enabling the identification of *cox1* haplotypes within individuals, with the advantage of a well-stocked *cox1* reference database that can eliminate contamination introduced during the experimental processes.

Since starvation can lead to sleep suppression, which causes the accumulation of ROS in fruit flies [8,9], and further considering that ROS can cause oxidative damage to mtDNA, here, by using high-throughput sequencing, we aimed to evaluate whether starvation would alter the levels of mitochondrial heteroplasmy, including the within-individual number of *cox1* haplotypes and genetic diversity, as well as the distribution of heteroplasmic sites in haplotypes. The results revealed that starvation, although had a limited impact on mitotype genetic diversity and mitochondria function, the effect on mitochondrial heteroplasmy may be gender-specific, implying that mitochondria of different gender may respond differently to starvation.

## 2. Results

### 2.1. The number of haplotypes and the average genetic distance of haplotypes within individual

**Figure 1.**
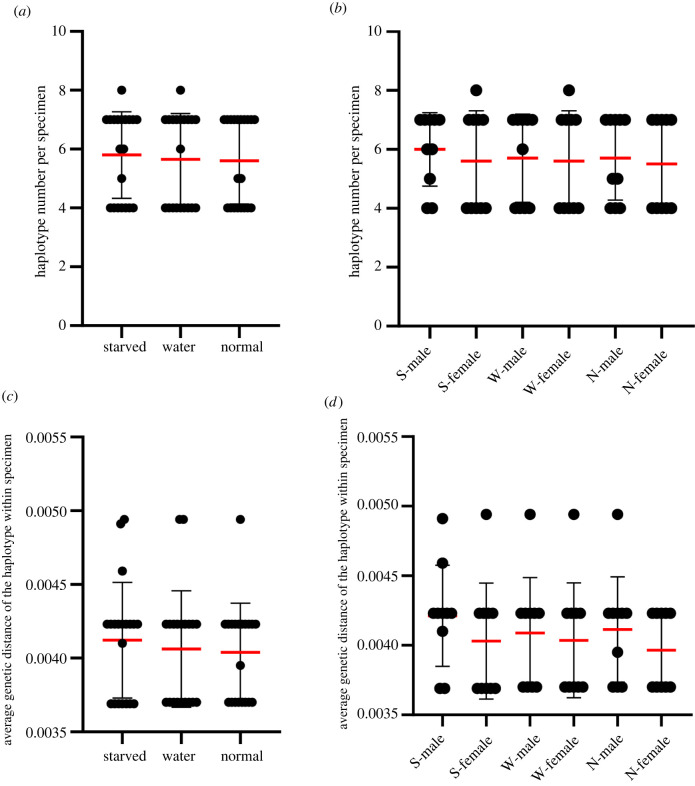
Comparison of the number of *cox1* haplotypes per specimen (*a*,*b*) and the average genetic distance of haplotypes within the specimen (*c*,*d*) among different groups: (*a*,*c*) all specimens; (*b*,*d*) females and males in the three treatments. Starved (‘S’ in *b*,*d*), water (‘W’ in *b*,*d*) and normal (‘N’ in *b*,*d*) indicate specimens in the three treatments. The red lines represent the average values, the black lines represent standard errors, the black circles represent the numbers of haplotypes (in (*a*,*b*)) as well as average genetic distances of haplotypes (in (*c*,*d*)) in each specimen.

Altogether 60 individuals in the three treatment groups were subjected to PCR amplification and high-throughput sequencing of the mitochondrial *cox1* gene fragment, and a total of 2 214 468 high-quality reads were obtained after quality control, with the sequencing depth of each sample from 49 814×to 90 509×(electronic supplementary material, table S1). After removing sequences with a frequency of less than 0.5%, the number of haplotypes in each individual ranged from four to eight, and the average genetic distance between haplotypes in each individual ranged from 0.369% to 0.494%, both values of which were higher in the starved group (figure 1*a,c*) than in the other two groups, nevertheless with no significant differences. In addition, from the comparison between males and females, regardless of the treatment group, the average number of haplotypes and the average genetic distances of haplotypes in the males were higher than those in the females (figure 1*b*,*d*).

### 2.2. Identification and comparison of major and minor haplotypes within individual

**Figure 2.**
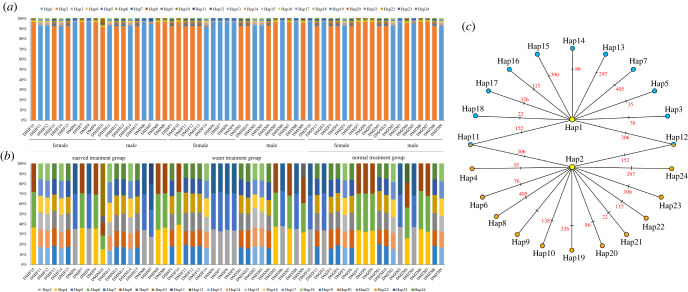
Stacked plot and network of haplotypes. (*a*) Proportions of all haplotypes detected in each individual and (*b*) relative proportions of all minor haplotypes (without Hap1 and Hap2) detected in each individual, with the horizontal axis showing the ID of different individuals, and the vertical axis presents the proportion of different haplotypes. The haplotypes are represented by different colours, as shown at the bottom; (*c*) the network of all the haplotypes was constructed based on sequence divergence. Yellow, blue and orange dots indicate different haplotypes, and the red numbers at the short lines on each long line represent the mutation sites between the haplotypes at both ends of the long line.

Twenty-four haplotypes (figure 2; electronic supplementary material, table S2) were identified, of which, two were randomly detected as the major (MAJ) haplotypes (Hap1 and Hap2). The MAJ haplotype sequences accounted for greater than or equal to 90% of the total sequences within individual (figure 2*a*; electronic supplementary material, table S2). In addition, no correlation was observed between the MAJ haplotypes and the feeding treatments, but the coexistence of these haplotypes was noted within individual (e.g. for DMJM7 and DMZM5, when the Hap1 was the MAJ haplotype, the Hap2 was the minor (MIN) haplotype, while for DMJM8 and DMSM9, when the Hap2 was the MAJ haplotype, the Hap1 was the MIN haplotype) (figure 2*a*; electronic supplementary material, table S2).

It was interesting to note that there was a correlation between the distribution of MIN haplotypes and the type of MAJ haplotypes within individual. When Hap1 was the MAJ haplotype, the MIN haplotypes were Hap3, Hap5, Hap7 and Hap13-Hap18; when Hap2 was the MAJ haplotype, the MIN haplotypes were Hap4, Hap6, Hap8 and Hap19-Hap24. The proportion of all the MIN haplotype sequences in the total sequences within individuals was very low (3.47%–8.76%; electronic supplementary material, table S3). After removing the sequences of the MAJ haplotypes, half of the individuals had three MIN haplotypes and the remaining had six MIN haplotypes (figure 2*b*). Furthermore, two unique MIN haplotypes (Hap10 & Hap11) were detected in the individuals of the starved group, a MIN haplotype of Hap9 was detected in some individuals of both the starved and water groups, and a MIN haplotype of Hap12 was found specifically in an individual of the normal group, all of which were only in males.

### 2.3. Analysis of heteroplasmic sites of *cox1* haplotypes within individual

For the heteroplasmic site identification, with all haplotype sequences of each individual as the dataset, we set its MAJ haplotype sequence as the reference sequence and defined the differential sites between the MAJ and MIN haplotypes as the heteroplasmic sites of the haplotype sequences contained in the individual. When comparing the heteroplasmic sites contained in each individual, regardless of the three feeding treatment conditions, there were eight heteroplasmic sites that could result in amino acid changes of the encoded proteins by non-synonymous substitutions (table 1; electronic supplementary material, table S4): C22T (Hap18 or Hap21), A35T (Hap5 or Hap4), G76A (Hap3 or Hap6), A115G (Hap16 or Hap22), G138A (Hap10), C297T (Hap13 or Hap24), T390A (Hap15 or Hap23) and T405G (Hap7 or Hap8).

**Table 1.** Variable sites of haplotypes distributed in specimens of the three treatments and PROVEN prediction results of the non-synonymous substitution sites. D, deleterious; N, neutral; —, synonymous mutation.

	Variable sites and PROVEN prediction results												
starved	C22T	A35T	G76A	T86C	A115G	C297T	T390A	A326G	T405G	A152G/G152A	C306T/T306C	G138A	A377G
normal	C22T	A35T	G76A	T86C	A115G	C297T	T390A	A326G	T405G	A152G/G152A	C306T		
water	C22T	A35T	G76A	T86C	A115G	C297T	T390A	A326G	T405G	G152A	T306C		A377G
PROVEN	D	D	D	—	D	D	N	—	D	—	—	N	—

In addition, there were two identical synonymous substitutions (152 and 306 sites) between the minor haplotype groups of Hap3 & Hap6, Hap4 & Hap5, Hap7 & Hap8, Hap13 & Hap24, Hap14 & Hap20, Hap15 & Hap23, Hap16 & Hap22, Hap17 & Hap19 and Hap18 & Hap21, and both of these synonymous substation sites were also the differential sites for the two major haplotypes (Hap1 and Hap2). According to the comparison of these differential sites, the relationship between different haplotypes was observed (figure 2*c*), revealing the pattern that all minor haplotypes were generated by mutations from the two major haplotypes.

**Figure 3.**
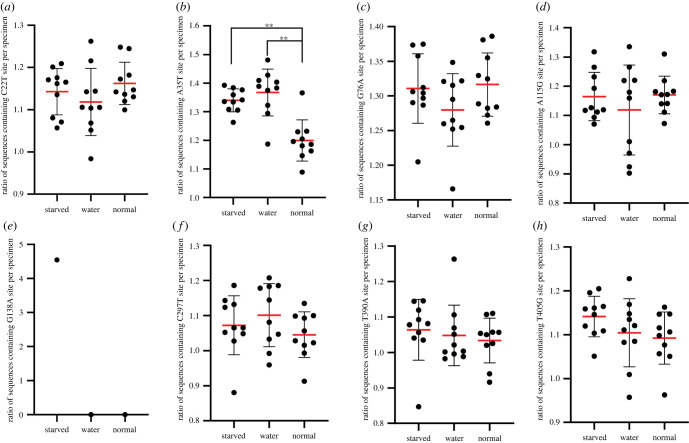
Analysis of the eight non-synonymous mutations shared by specimens. The horizontal axis shows the three treatments, and the vertical axis represents the ratio of sequences containing each non-synonymous site per specimen (*a*–*h*). Notes: the red lines represent the average values, the black lines mean standard errors, and the black circles mean the numbers of sequences containing different mutation sites in each specimen. Double asterisk (**) indicates significant difference (*p*-value < 0.01).

The possible effects of the eight non-synonymous substitutions on protein function were predicted by the PROVEN Protein program, and the results showed that six of them were deleterious substitutions. By further comparing the average proportion of haplotype sequences containing these eight non-synonymous substitution sites in the total sequences of individuals among the individuals of the three different treatment groups (figure 3), it was found that individuals in both the starved and water groups contained a significantly higher average content of haplotype sequences containing the heteroplasmic site of A35T than those in the normal group (figure 3*b*). Besides, a shared synonymous mutation (A377G) was screened out in individuals of both the starved and water groups, which was only detected in one individual in each of the two treatment groups (DMJM10 and DMSM9; electronic supplementary material, table S5), and a non-synonymous substitution (G138A; electronic supplementary material, table S5) occurred in the individual of DMJM10 in the starved group, with PROVEN Protein predicted as a neutral substitution (table 1).

### 2.4. The reactive oxygen species levels within individual

Among the three feeding treatments, the level of ROS in the starved group was significantly lower than in the other two groups. The water and normal groups had similar levels of ROS, with the normal group having a slightly higher level (electronic supplementary material, figure S1).

## 3. Discussion

In this study, the correlation between starvation and the haplotype genetic diversity of mitochondrial *cox1* gene fragment was investigated by using laboratory-reared adult *Drosophila melanogaster*. The result indicated that the coexistence of two mitotypes is present in the reared fruit fly population, which is not uncommon in natural or laboratory-reared populations [48–51], and several hypotheses have been proposed to explain the maintaining mechanisms of the coexistence of two mitotypes within populations, such as negative frequency-dependent selection (NFDS), direct natural selection of mtDNA and cytonuclear coadaptation [52]. Among them, NFDS played a role in maintaining the coexistence of multiple mitotypes in the population of *Drosophila subobscura*, and food conditions could enhance this selective effect [53]. We thought that the coexistence of two mitotypes in *D*. *melanogaster* in this study might also be due to NFDS.

The results of the study revealed an association between starvation and the heteroplasmic sites of mitotypes in individual adults of *D*. *melanogaster*, although the sequences containing these heteroplasmic sites might not have a significant deleterious effect on organismal function due to their low proportion. Firstly, the results of the distribution pattern of MAJ and MIN haplotypes and the origin of the heteroplasmic sequences suggested that the heteroplasmic sequences we detected were not related to the random errors during PCR and amplicon sequencing, rather they were truly non-random mutation sites. Subsequently, although six of eight non-synonymous substitutions were deleterious, the proportions of these mutation-containing sequences were low and given that deleterious mutations in mitochondria would not show corresponding symptoms in the organism until they had accumulated beyond a certain proportion [54,55], the presence of these deleterious mutations should not have too many deleterious effects on the organism. Moreover, the significantly higher levels of mutation site A35T in the starved and water groups implied that starvation could increase the content of haplotype sequences containing some specific mutations within individuals, possibly related to the stress of starvation imposed on the fidelity of mtDNA.

Male fruit flies have a higher level of mitochondrial heteroplasmy than females, and starvation could further increase the level of heteroplasmy in males. In bivalves, the males may possess specific sequences derived from the male parent through the doubly uniparental inheritance [56,57], resulting in higher mitochondrial heteroplasmy in males than females; among insects, some male fig wasps (Hymenoptera, Insecta) also showed a higher level of heteroplasmy [45], which suggests that a higher level of heteroplasmy in males than in females may be a more general phenomenon. In this study, we not only revealed a higher level of mitochondrial heteroplasmy in male fruit flies than in females but also found a gender-specific increasing effect of starvation on mitochondrial genetic diversity, with the increasing effect higher in males.

Of all the detected haplotypes, the vast majority were shared among the three treatment groups except only two unique haplotypes (with A377G, synonymous substitution and G138A, non-synonymous substitution) in the starved group. Subsequently, the threshold of haplotype sequence screening was relaxed (changing from ‘removing the sequences whose frequency is lower than 0.5% of the total sequences’ to ‘removing only singletons which may be caused by errors during PCR and sequencing’ to detect whether these two haplotypes were also present in other individuals). As a result, both haplotypes could be identified in some individuals of the three treatment groups. Therefore, the occurrence of these two unique haplotypes was only due to the strict screening threshold, which likewise suggested that starvation might increase the abundance of these two haplotype sequences, making them easier to be screened out in the individuals of the starved group. Of course, it was worth mentioning that the contingency caused by the PCR process was also a factor that cannot be ruled out.

We further explored the association between the levels of ROS and the increased mitochondrial heteroplasmy by starvation in fruit flies. However, the ROS levels showed a pattern completely different from previous studies [8,9]. This apparently different association between ROS levels and starvation detected in our and previous studies may come from the different sample handling strategies. In the previous studies, levels of ROS in the dorsal fan-shaped body of the brain [8] or the gut [9] were measured separately, while here used the whole-body homogenates. The reason we chose this process was that the mitochondrial heteroplasmy data we obtained came from the whole body, so the ROS levels should also be measured for the whole body. Although starvation may result in lower ROS levels in the whole body, it could still cause damage to mtDNA given the elevated ROS levels in some tissues. Consequently, in the future, we will focus more on the relationship between starvation and levels of ROS and mitochondrial heteroplasmy in different tissues.

## 4. Materials and methods

### 4.1. Breeding of the fruit flies and sample preparation

The *D. melanogaster* population (which was kindly donated by Prof. Hu Haoyuan in Anhui Normal University) used in this experiment had already been reared in our laboratory for at least 50 generations after tetracycline treatment to remove *Wolbachia* infection [58]. The third instar pupae were placed in transparent plastic cups, covered with gauze at the rims for ventilation and reared in an artificial climate box (26°C±1°C, 60% humidity, 7000 lx of light, and 14 : 10 h light: dark cycle). Pupal hatching was observed daily and when the first adult emerged, the adults were collected once a day, then randomly and equally transferred to three plastic cups configured with different food: (i) normal feeding group—standard corn flour medium for fruit flies were provided; (ii) water supply group—only distilled water were provided; (iii) starved group—no food and water was provided. The individuals that had been placed in different food configurations for 2 days were taken out and put in absolute ethanol separately and stored at −20°C.

### 4.2. DNA extraction, PCR and sequencing

Ten males and 10 females were randomly selected from each treatment group and washed twice with distilled water to remove the absolute ethanol. According to the instructions, the total DNA of each specimen was extracted from the whole body using *EasyPure*® Genomic DNA Kit (TransGen, Beijing, China) and stored at −20°C after quality inspection by using NanoDrop (Thermo Fisher Scientific).

For standard Sanger sequencing, the PCR reaction mixture was 25 µl for the amplification of *cox1* gene per specimen, consisting of 0.5 µl (10 µM) each of forward (MiniLepF1, 5′-GCTTTCCCACGAATAAATAATA-3′) [59] and reverse (HCO2198, 5′-TAAACTTCAGGGTGACCAAAAAATCA-3′) [37] primers, 2.5 µl 10 ×TransTaq HiFi buffer, 2 µl dNTP, 0.25 µl TransTaq DNA polymerase high fidelity (TransGen, Beijing, China), 1 µl template and 18.25 µl PCR water, with thermocycling profiles as follows: 94°C for 5 min; 35 cycles of 94°C for 30 s, 51°C for 30 s, and 72°C for 45 s; and 72°C for 7 min. A 3.5 µl of PCR products were used for electrophoresis on 1% agarose gel and the positive PCR products were bi-direction sequenced (Songon Biotech, China).

For amplicon sequencing, an additional 6 bp barcode sequence was added to the 5′ end of both primers (MniLepF1 & HCO2198). Each individual was set-up with three PCR replicates and the PCR reaction mixture was 25 µl, including 3 ng of total DNA, 12.5 µl 2× Q5 High-Fidelity Master Mix (New England Biolabs, Ipswich, MA, USA), 1.25 µl (10 µM) of both forward and reverse primers and the rest supplemented with PCR water. The thermocycling profiles were 98°C for 30 s; 35 cycles of 98°C for 30 s, 51°C for 30 s, and 72°C for 30 s; and 72°C for 2 min. A 3.5 µl of PCR products was used for electrophoresis on 1% agarose gel, and the three positive PCR products of the same specimen were mixed and purified using MagicPure Size Selection DNA Beads II (TransGen, Beijing, China). A DNA library was constructed by mixing PCR products of 10 specimens and performed VAHTS Universal Pro DNA Library Prep Kit of pair-end Illumina Miseq (2 × 300) sequencing (Genewiz, Suzhou, China).

### 4.3. Data analysis

Sanger sequences for the *cox1* gene were aligned and trimmed by BioEdit using the ClustalW algorithm [60] with default parameters to a final length of 407 bp, and frameshifts or nonsense mutations were then checked using EditSeq to exclude numts. For high-throughput data processing, nine steps were performed (electronic supplementary material, S1), obtaining haplotypes after removing sequences with frequencies lower than 0.5% of the total reads, and in each individual, the haplotype with the largest number of reads was called major haplotype, while all of the rest haplotypes were called minor haplotypes [45].

The average genetic distance of haplotypes within each specimen was calculated by MEGA 10 [61]. We used all haplotype sequences within each individual to analyse the heteroplasmic sites, by setting the major haplotype as the reference sequence to search for the differential sites between major and minor haplotypes. DnaSP 6.0 [62] was used to identify the same haplotypes among different specimens. The effect of non-synonymous substitution on protein function was predicted by the online program PROVEN Protein (http://provean.jcvi.org/seq_submit.php). A median-joining network was constructed based on the haplotype sequences of Hap1-Hap24 using Network 5.0.1.0 [63]. The SPSS v20 was used to analyse the statistical differences in the data, and the independent samples *t*-test was performed for data that met the normal distribution; those data that did not meet the normal distribution were examined using the Mann–Whitney *U*-test.

## Data Availability

The high-throughput sequencing data of all individuals in the three treatment groups have been deposited in GenBank under the accession ID of BioProject PRJNA761665. The data are provided in the electronic supplementary material [64].
